# Comparative Study about Dimensional Accuracy and Surface Finish of Constant-Breadth Cams Manufactured by FFF and CNC Milling

**DOI:** 10.3390/mi14020377

**Published:** 2023-02-02

**Authors:** Enrique E. Zayas-Figueras, Irene Buj-Corral

**Affiliations:** Department of Mechanical Engineering, Barcelona School of Industrial Engineering (ETSEIB), Universitat Politècnica de Catalunya (UPC), Av. Diagonal, 647, 08028 Barcelona, Spain

**Keywords:** constant-breadth cam, design process, FFF, CNC milling, dimensional accuracy, surface finish

## Abstract

In this work, the design, manufacture and measurement process of constant-breadth cams is presented. The motion law of the cam was designed by means of Bézier curves and the corresponding design desmodromic constraints. The cams were manufactured in two different materials employing two different processes: PLA cams with fused filament fabrication (FFF) and aluminium cams with computer numerical control (CNC) milling. The main aim of this work is to compare both types of cams regarding dimensional accuracy and surface finish, in order to evaluate if it would be possible to temporally replace a metallic cam with a plastic one during the repair of the first one. Dimensions were measured with micrometres and surface roughness with a contact roughness meter. The results show that, in diametral dimensions, similar dimensional error values were obtained for both the 3D-printed and the machined cams. However, in longitudinal dimensions, whose direction is perpendicular to the 3D-printed layers, the 3D-printed cams showed higher dimensional error than the machined ones. The average roughness *R*_a_ in the 3D-printed cams was 20 times higher than in the milled cams. According to the results, it would be recommended to temporally replace metallic cams with plastic ones in applications of low-power transmission. Given that in the literature little information is available about the measurement of 3D-printed desmodromic cams, this work will contribute to the study and analysis of this kind of 3D printed mechanism.

## 1. Introduction

**Figure 1:**
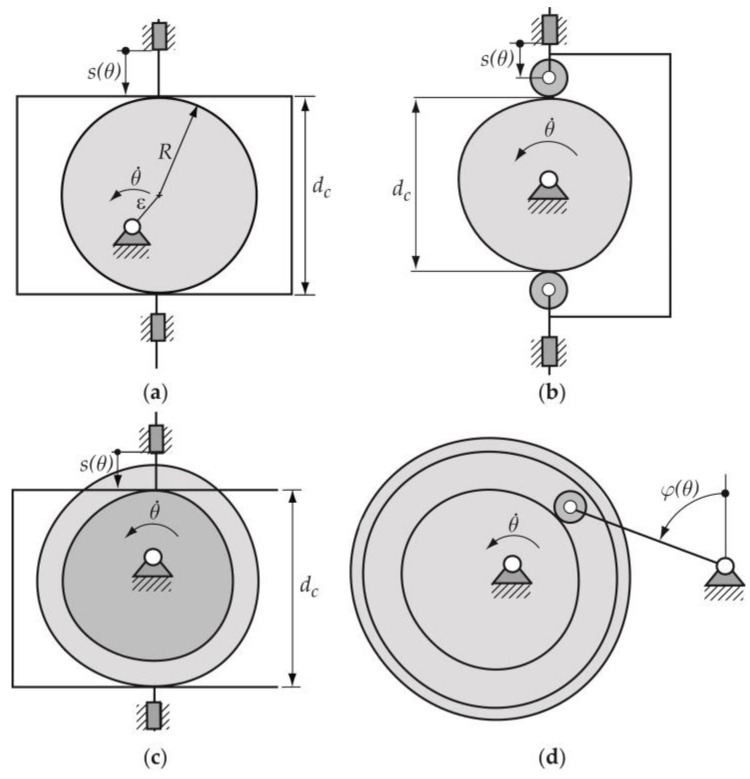
Types of planar desmodromic cam mechanisms: (**a**) constant-breadth cam; (**b**) constant-diameter cam; (**c**) conjugate cams; and (**d**) slotted cam mechanisms, respectively.

**Figure 2:**
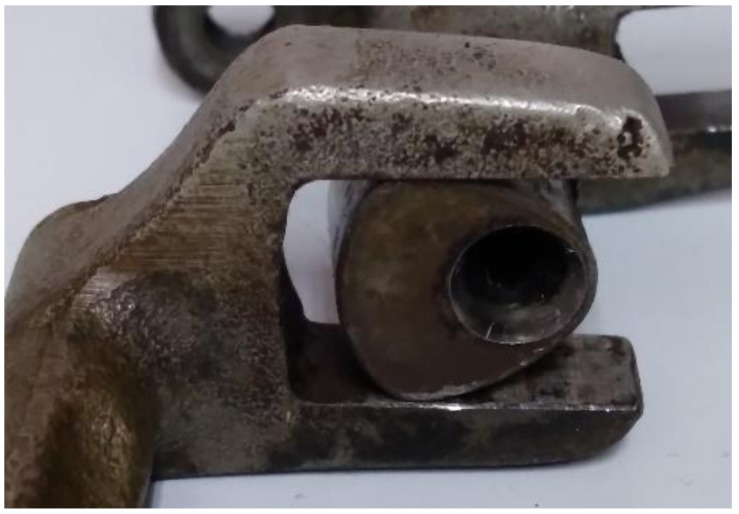
Photograph of the constant-breadth cam mechanism with a flat-faced double-oscillating follower, used in a traditional sewing machine.

Desmodromic cam mechanisms use two upper conjugate pairs (geometric closure of the upper pairs) in such a way that both impose the same kinematic restriction, guaranteeing global bilaterality: the loss of contact in one of the pairs is prevented by the other pair. There are four types of planar desmodromic cam mechanisms: (a) constant-breadth cam mechanism, (b) constant-diameter cam mechanism, (c) conjugate cam mechanism and (d) slotted-face cam mechanism (Figure 1). These types of mechanisms are used in different industries such as textile, automotive, etc. Figure 2 shows a photograph of a constant-breadth cam mechanism employed in a traditional sewing machine.

In the case of constant-breadth cam mechanisms, here exposed, the geometry of both cam and follower guarantees the closure of the higher kinematic pairs formed by the bilateral contact of both parts; and this fact implies the precise design and manufacture of the profile of both the cam and the follower. The constant-breadth cams may either be circular arc cams or have arbitrary geometry [1]. An eccentric cam that drives a parallel flat-faced double follower (Figure 1a) can be considered the simplest example of a constant-breadth cam, due its profile being a circumference of radius *R*. In this mechanism, the diameter 2*R* of the eccentric cam coincides with the constant distance *d*_c_ between the two parallel flat surfaces of the follower [2]. The double follower can have a translating or oscillating movement; nevertheless, the displacement function of the follower that guarantees obtaining the corresponding constant-breath cam profile is subject to certain restrictions and consists of two segments: the first one called the designed segment, and the second, which is calculated from the designed segment, called the calculated segment [2,3,4].

The design process of a cam follower mechanism regarding the geometric and kinematics aspects once the starting design parameters have been chosen consists of three stages: the first stage, the design or synthesis of the follower displacement function—also known as displacement law; the second stage, obtaining the cam profile that corresponds to the motion law before it is obtained; and the third stage, verifying the correctness of the cam profile geometry by means of evaluating its radius of curvature to avoid an incorrect contact between the cam and the follower. In a previous study [4] conducted by one of the present study’s co-authors, the direct synthesis method of a constant-breadth cam mechanism with a parallel flat-faced double-translating follower was presented, showing numerical examples. The design process used in the present work is based on this method. 

The methodology used in the present work consists of three main parts: (1) the cam–follower mechanism design process; (2) the manufacturing processes employed to obtain the constant-breadth cam profile by filament fused fabrication (FFF) and numerical control milling processes; and (3) the measurement of the cam profiles obtained—dimensional errors and surface finishing. The mentioned methodology was applied to a constant-breath cam mechanism that drives a translating flat-faced double follower. In the first part of the methodology—the design process—once the designer agrees with the cam profile obtained in 2D (two dimensions), a 3D (three dimensions) virtual model of the cam–follower mechanism is obtained using computer aided design (CAD) software, where a simulation of the performance of the mechanism is carried out. Later, a prototype of the mechanism is obtained using the additive manufacturing process above mentioned; the physical parts available are checked and later measured. This kind of mechanism is very important to measure the dimensional errors and the surface finish of the cam, and a principal dimension to be checked is the breadth of the cam *d*_c_ (Figure 1a).

The usual manufacturing methods for cams include forging, casting, milling, etc. For example, Lugosi et al. [5] assembled forged powder cams to a shaft by brazing, in order to obtain a camshaft. Luis Perez et al. [6] studied the wear of magnesium aluminium alloy isothermally forged cams. They found a better performance in cams subjected to equal channel angular pressing (ECAP). Nowadays, camshafts are obtained in nodular iron by computer numerical control (CNC) [7]. Forged steel camshafts allow higher loads than cast ones. However, they require subsequent machining processes such as deep drilling to reduce their weight. An alternative way to reduce weight consists of using composite camshafts, which are composed of two different materials and, subsequently, assembled. Typically, a steel tube is used in which the forged cams are assembled: for example, with a shrink fit. In recent years, the lost foam-casting process has been used to produce steel camshafts with hollow shapes that are lighter than the conventional ones. The final machining stage of a camshaft is often a grinding process [8]. In a previous work, the authors of the present paper manufactured flank cams by milling and electro discharge machining (EDM). EDM has the advantage of enabling parts in different conductive materials to be obtained [9]. Some authors have obtained and applied cam–follower mechanisms through additive manufacturing; for example, Cheng et al. [10,11] evaluate the kinematic performance of a desmodromic cam mechanism (a particular type of mechanism that they called composite cam–follower mechanism) that is used to obtain personalized automata. Another application of the 3D-printed cam–follower mechanism is a micro-cam-actuated linear peristaltic pump for microfluidic applications, presented by Xiang et al. [12]. Almeida et. al. [13] present the design and modelling of a 3D-printed flexure-based actuation mechanism for robotic microtweezers. The key component is a uniquely designed cam flexure system 3D printed in nylon, which linearly translates the bending of a piezoelectric bimorph actuator into angular displacement. The authors stated that 3D printing made it possible to manufacture the cam with a specifically calculated curve, which would otherwise have been costly using conventional milling techniques.

Different methods are used to measure the dimensions of cams, which can be divided into two groups: non-contact methods (reflection, laser interferometry, electromagnetic induction) and mechanical contact methods [14]. As for non-contact methods, for example, vision machines are used to measure the cam distance in camshafts [15]. A laser sensor is often employed to measure complex shapes, such as those of the cams [16]. In another example about the fabrication of complex shapes, such as those of dental prostheses, Jeong et al. [17] used the DLP (digital light processing) additive manufacturing process and the CNC milling process to compare the dimensional accuracy achieved by both methods. The prostheses, made of dental resin, were measured by laser scanning, and it was observed that the error of the CNC fabricated parts was higher than that of the DLP printed parts. On the other hand, electromagnetic induction, for instance, is used to determine the angular rotation of rotative components such as gears [18]. A typical contact measuring device for cams is the five-axis coordinate measuring machine [19]. However, conventional measurement tools can also be employed for the same purpose [20].

In the literature, there is little information about the dimensional accuracy and surface finish of 3D-printed cams. In the present work, both the dimensions and roughness values of constant-breadth cams are presented. They were manufactured using both the fused filament fabrication (FFF) and the milling processes. The main reason for the comparison is the possibility of temporarily replacing the metallic cams with those made of PLA obtained by FFF, while the metallic ones are repaired in mechanisms that have more of a kinematic type of operation than high-power transmission. Moreover, the 3D-printed cam–follower mechanism can also be used in scale models in the field of engineering education.

## 2. Materials and Methods

**Figure 3:**
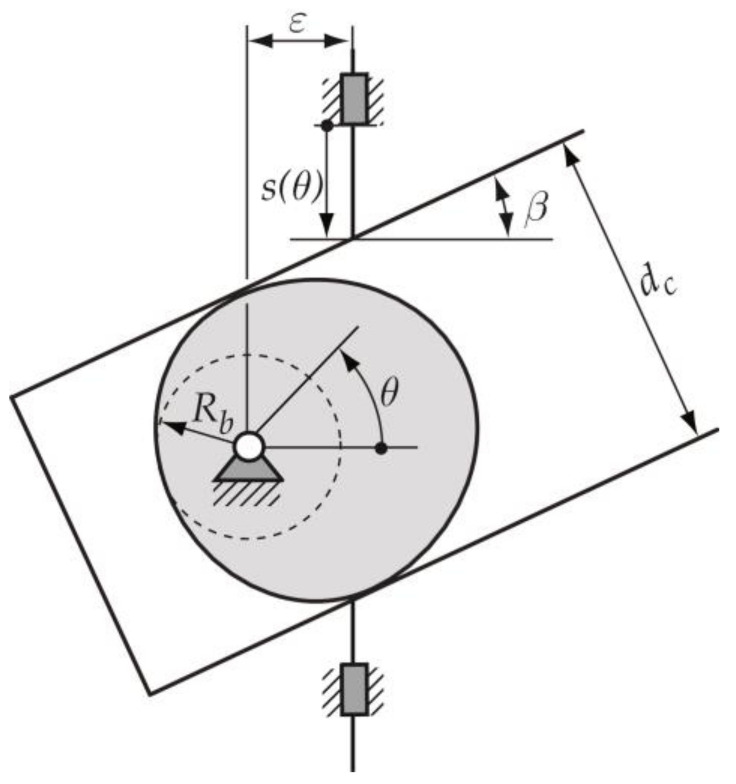
Geometric parameters in a constant-breadth cam mechanism: $R_{b}$, radius of the base circle of the cam; *ε*,  offset of the follower; *β*, inclination angle of the translating follower; and $\;d_{c},$ the breadth of the cam.

The cam mechanism exposed in this work is a constant-breadth cam with a double flat-faced translating follower, whose geometric parameters are presented in Figure 3. Thus, this section introduces the design process applied to obtain and verify the adequate constant-breadth cam profile, from which the 3D model of the cam is obtained. The manufacturing and measuring processes are also explained.

### 2.1. Design Process of the Cam–Follower Mechanism

Once the designer has chosen the type of cam mechanism to be designed (a constant-breadth cam with a double flat-faced translating follower), the starting data for the design process must be established. These data are: (a) the radius $R_{b}$ of the base circle of the cam; (b) the breadth of the cam $d_{c}$, which coincides with the distance between the two parallel flat surfaces of the follower; (c) the offset ε of the translating follower; and (d) the inclination angle β of the translating follower (Figure 3). Table 1 shows the values of the data here used.

In a cam–follower mechanism the cam drives the follower according to the desired follower displacement law: s(θ), as a function of the angle θ rotated by the cam. The design sequence of the cam mechanism, as mentioned in Section 1, consists of the following steps that are explained for the particular case here exposed [2,4].

1. *Designing the displacement law*s(θ). The designer defines the displacement law of the follower according to the movement required by the technological process or function that must be met by the mechanism. As mentioned in Section 1, the displacement law for this particular type of constant-breadth cam mechanism with a double-translating follower must meet a design restriction (expression 1) [2,3,4]. Thus, s(θ) consists of two segments: the designed segment and the calculated segment.

(1) $$s\left(\theta \right)+s\left(\theta +180{}^{\circ}\right)=\;\mathrm{constant}=d_{c}$$

**Figure 4:**
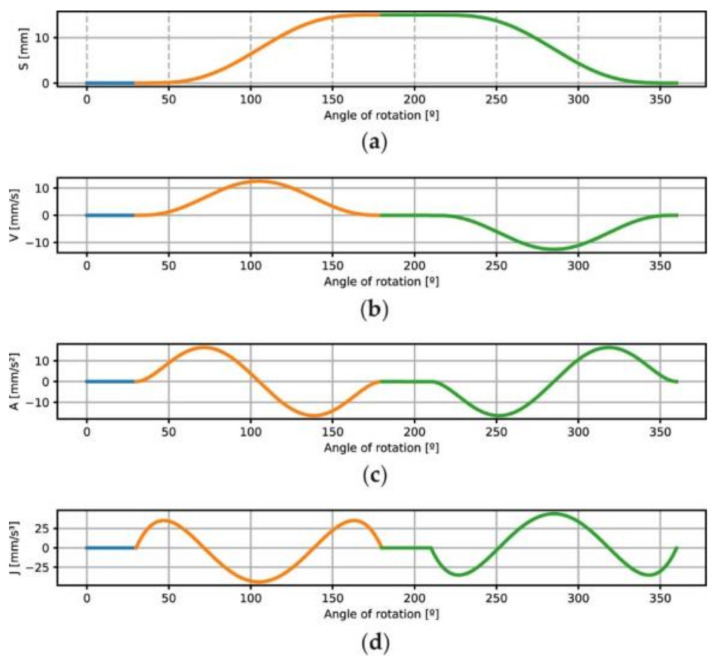
(**a**) The designed displacement law s(θ) and its derivatives: (**b**) the velocity curve V(θ), (**c**) the acceletarion curve (θ) and (**d**) the jerk curve J(θ).

In the case here exposed, the design segment is defined in the interval θ∈[0, 180°] using a non-parametric Bézier curve with continuity $C^{3}$, and it comprises one dwell event during 30° of cam rotation and one rise event from 30° to 180°. The desired follower maximum displacement $s_{\max}\left(\theta \right)\;$ is 15 mm. The use of free software, called QtCAM [21], is used to support the design process. For the calculated segment in which θ∈[180°, 360°], it is synthesized according to the process exposed in [4], as well as consisted of two events: an upper dwell of the follower while the cam rotated from 180° to 210°, and a fall event until completion of the entire cycle, 360°. Figure 4a shows the entire displacement law s(θ) obtained and its corresponding derivatives: the first is the velocity function V (θ) (Figure 4b), the second is the acceleration function A(θ) (Figure 4c) and the third is the jerk J(θ) (Figure 4d), which allows the designer to make decisions regarding the process design. This guarantees the good performance of the mechanism. In the graphs (Figure 4), the corresponding events of motion are drawn in different colours, with green corresponding to the calculated segment.

2. *Obtaining the cam profile* that drives a certain type of follower according to the designed displacement law.

**Figure 5:**
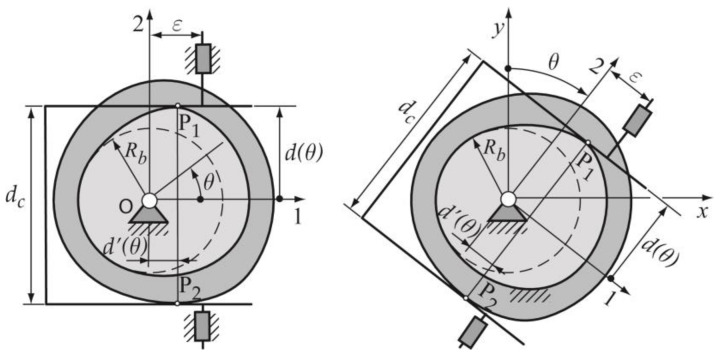
Conjugate cam mechanism and its kinematics inversion.

The method applied to obtain the cam profile is based on the proposal of Zayas [22], in which a constant-breadth cam is obtained from the method of generating a mechanism of conjugated cams (Figure 5), where each of them has contact with the flat faces of the double-translating follower; the first cam contacts with the upper point $P_{1}$, and the second cam contacts with the lower point $P_{2}$. The two conjugated cams must have the same geometry and the same orientation, so that they coincide and overlap to represent a single cam of constant breadth $d_{c}$ between the parallel faces of the double follower. The profiles of the conjugate cams are obtained from the parametric expressions (2)–(5). The analytic–vectorial method to obtain the cam profile is based on a kinematic inversion, in which two coordinate systems are used, one (*x*, *y*) in which the cam is fixed, and the other (1, 2) fixed and oriented according to the follower guide (Figure 5).

The parametric expressions of the position vectors of the contact points $P_{1}$ and $P_{2}$ in the mobile base 1, 2 (Figure 5) are the expressions (2) and (3), and in the fixed base x, y are the expressions (4) and (5); these last expressions are required to obtain the cam profiles.

(2) $${\left\{\bar{OP_{1}}\left(\theta \right)\right\}}_{1,2}={\left\{\begin{array}{c} d_{1}^{\prime }\left(\theta \right)\\ d_{1}^{}\left(\theta \right) \end{array}\right\}}_{1,2},\;\;\;\;d_{1}^{}\left(\theta \right)=d_{o}^{}+s\left(\theta \right)\;\;\;\;\;$$

(3) $${\left\{\bar{OP_{2}}\left(\theta \right)\right\}}_{1,2}={\left\{\begin{array}{c} d_{2}^{\prime }\left(\theta \right)\\ d_{2}^{}\left(\theta \right) \end{array}\right\}}_{1,2},\;\;\;\;d_{2}^{}\left(\theta \right)=\left(d_{o}^{}-d_{c}\right)+s\left(\theta \right)\;\;\;\;\;$$

(4) $${\left\{\bar{OP_{1}}\left(\theta \right)\right\}}_{x,y}=\left[S_{\theta }\right]{\left\{\begin{array}{c} d_{1}^{\prime }\left(\theta \right)\\ d_{1}^{}\left(\theta \right) \end{array}\right\}}_{1,2},\;\;\;\;\left[S_{\theta }\right]=\left[\begin{array}{cc} \cos\theta & \sin\theta \\ -\sin\theta & \cos\theta \end{array}\;\right]\;\;$$

(5) $${\left\{\bar{OP_{2}}\left(\theta \right)\right\}}_{x,y}=\left[S_{\theta }\right]{\left\{\begin{array}{c} d_{2}^{\prime }\left(\theta \right)\\ d_{2}^{}\left(\theta \right) \end{array}\right\}}_{1,2}\;\;$$

In expressions (2) and (3), the term $d_{o}\;$ is the minimum distance between the follower and the centre of rotation of the cam up to the point where the follower starts to move. In expressions (4) and (5), $\left[S_{\theta }\right]$ is the rotation matrix.

Thus, to obtain a constant-breadth cam profile, in addition to the requirement of displacement law (expression (1)), another design constraint must be met, which is that the value of the constant-breadth cam $d_{c}$ should be [2,4,21]:

(6) $$d_{c}=2R_{b}+s_{max}\left(\theta \right)$$

**Figure 6:**
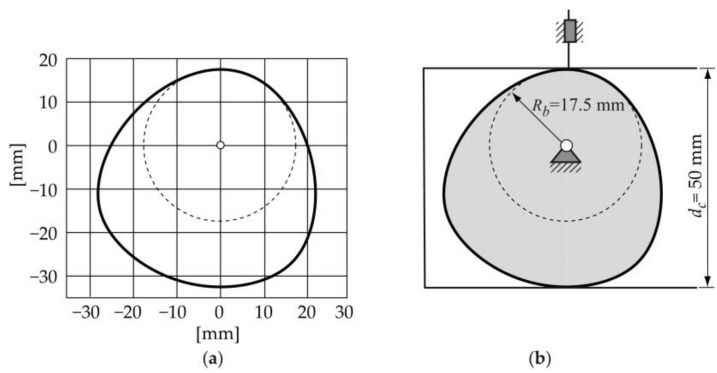
(**a**) Cam profile obtained, (**b**) constant-breadth cam mechanism.

Figure 6 depicts the constant-breadth cam profile obtained for the case here shown with the parameters shown in Table 1 and the displacement law exposed in Figure 4.

Once the cam profile is obtained, the third step of the design process is as follows.

3. *Verifying that the cam profile obtained* does not present geometric characteristics that prevent the right contact between the cam and follower. In the case here exposed, the curvature radii $r_{c}$ of the cam profile must always be positive, in order to guarantee correct contact between the cam and the follower. The expression to calculate $r_{c}$ is as follows [1]:
  (7) $$r_{c}\left(\theta \right)=\left(s\left(\theta \right)+s_{\theta \theta }\left(\theta \right)\right)\cos\beta -\varepsilon \sin\beta$$

**Figure 7:**
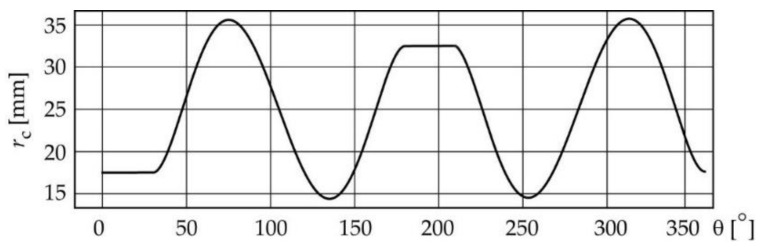
Curvature radii $r_{c}$ of the cam profile with *ε* = 0 and *β* = 0.

Figure 7 shows the graphics of the curvature radii $r_{c}$ corresponding to the constant-breadth cam profile obtained (Figure 6).

**Figure 8:**
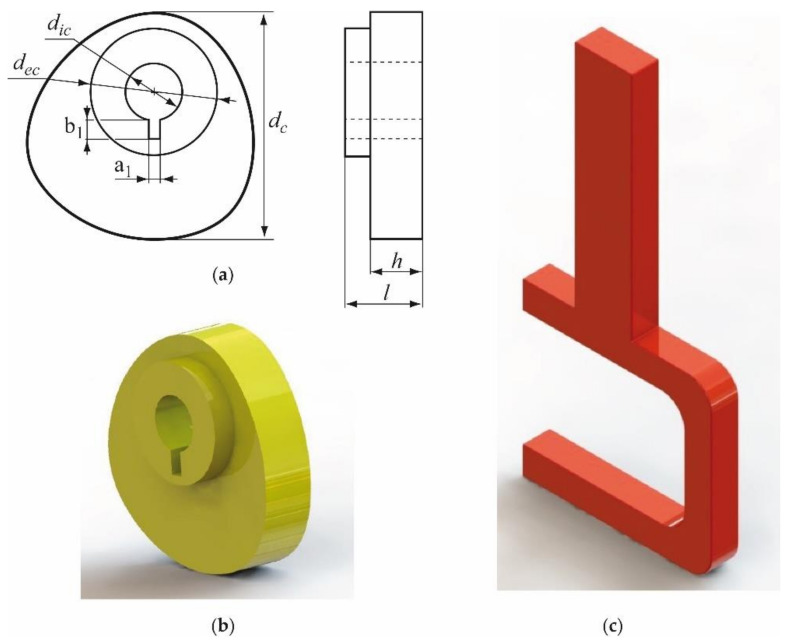
(**a**) Main dimensions of the hub and keyway of the cam, and virtual models of the (**b**) constant-breadth cam and (**c**) the double flat-faced follower.

In this particular case, the inclination angle of the translating follower is β = 0 (Table 1). This angle coincides with the pressure angle, which is an index of the quality of the mechanism; therefore, it is equal to zero, which is an advantage for the dynamic performance of the mechanism because the force exerted by the cam to drive the follower is always vertical. Thus, the next design step is to obtain the 3D virtual design of the cam–follower mechanism and to validate the design. Some additional geometrical dimensions (theoretical dimensions) shown in Figure 8a are defined to obtain the 3D virtual model of the cam. These dimensions are: the internal and external diameters of the hub of the cam *d*_ic_ = 12 mm and *d*_ec_ = 26 mm, respectively; the dimensions of the keyway, *a*_1_ = 3 mm and *b*_1_ = 5.19 mm; the width of the working face of the cam, *h* = 12 mm; and the total length of the cam, *l* = 15 mm. These values were defined, taking as a reference the dimensions of the shaft where the cam was to be placed. Using the CAD software SolidWorks, 3D virtual models were created of all the parts of the mechanism (the cam (Figure 8b), the follower (Figure 8c), the shaft of the cam, the frame of the mechanism, etc.).

**Figure 9:**
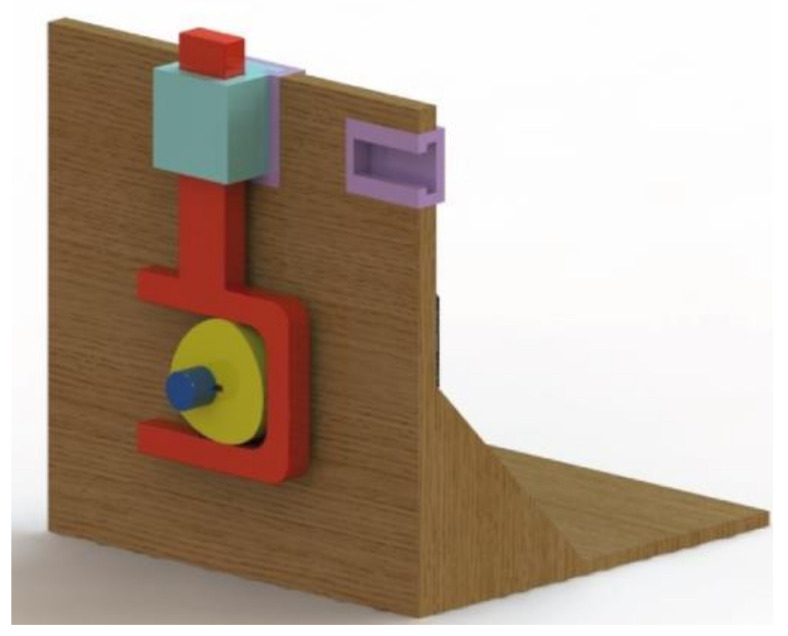
A virtual model of the assembled cam–follower mechanism.

**Figure 10:**
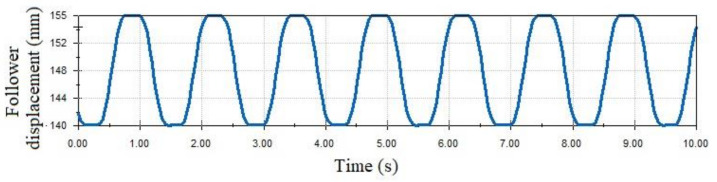
Follower displacement function, for several operating cycles, obtained with the motion module of SolidWorks.

Figure 9 depicts the assembly of the cam–follower mechanism, and Figure 10 shows the curve of the follower displacement obtained by means of the module motion of SolidWorks. The mentioned curve was obtained for several operating cycles, and it can be seen that, for a single work cycle, the events of the follower motion consist of two dwell segments, a rise segment and a fall segment, which coincide with the program of the motion law that was designed (Figure 4a), thus validating the correctness of the design.

Once the constant-breadth cam–follower mechanism design is considered correct, the next step is to manufacture the parts of this mechanism, to measure and check them, as well as to perform the assembly of the mechanism. Following this, the manufacturing process is presented.

### 2.2. Manufacturing Processes of the Cams

The designed cams were manufactured following an additive manufacturing process (3D printing) to obtain the prototypes and, as well as the milling process, to obtain the metal cams.

#### 2.2.1. Manufacturing Processes of the 3D-Printed Cams

The 3D-printed cams were obtained using the fused deposition modelling (FDM) or fused filament fabrication (FFF) technique. An Ultimaker 2+ machine was employed, with a 2.85 mm diameter polylactic acid (PLA) filament from the manufacturer RS PRO (particularly the material with RS code 832-0270). The main printing parameters, which were established by employing the slicer software Ultimaker Cura, are summarized in Table 2.

**Figure 11:**
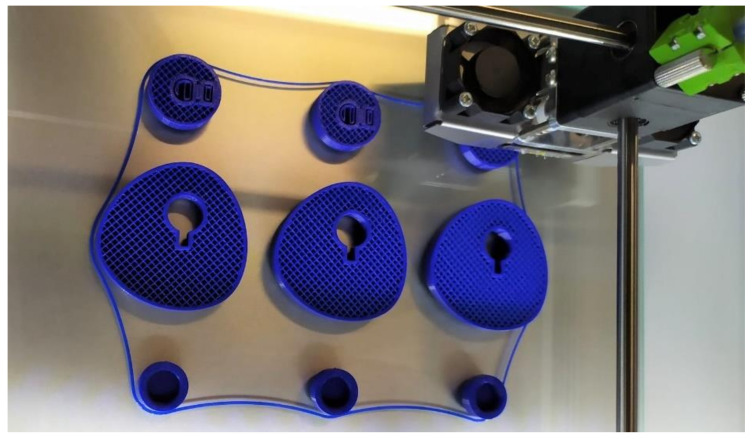
Constant-breadth cams being printed on the bed of the printer Ultimaker 2+.

Figure 11 shows an image of the cams while being printed on the bed of the Ultimaker 2+ printer. These parts are the prototypes of the cams, which are measured later.

#### 2.2.2. CNC Milling of the Cams

**Figure 12:**
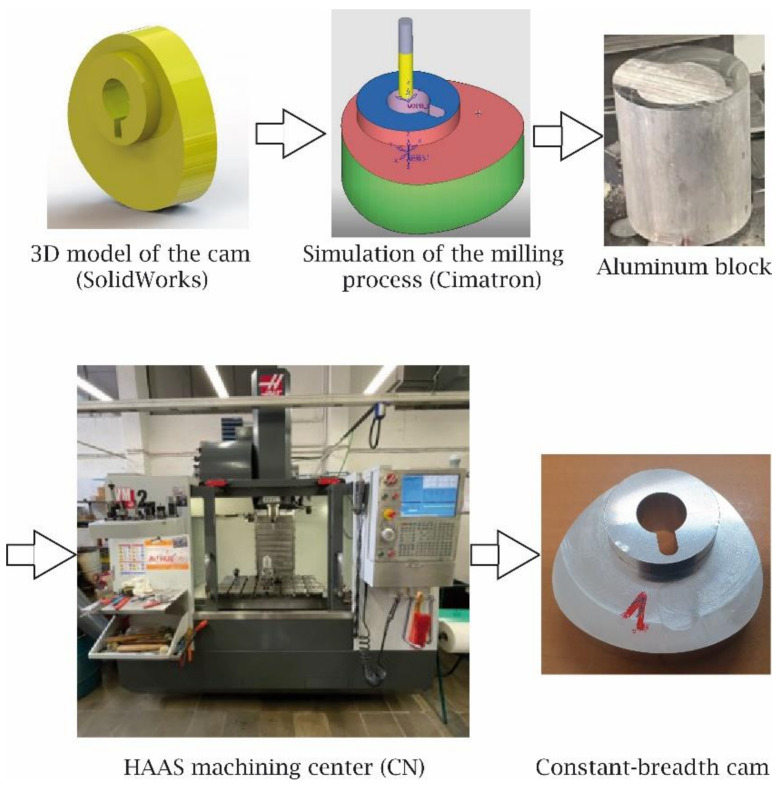
The sequence of steps followed to manufacture the metallic cams.

Once the prototypes of the cam were validated, it was decided to manufacture three metallic cams through the CNC milling process. First, using the 3D model of the cam obtained with the CAD SolidWorks, a manufacturing simulation process was carried out by means of the CAD-CAM software Cimatron 11 from Cimatech [23]. This simulation process allowed to validate the machining strategies, the tools and the machining parameters to be used, as well as the corresponding G-code to be used in the Hass VM2 3-axis machining centre. The metal cams were obtained from an aluminium block. Figure 12 shows the sequence of steps followed for the manufacturing process of the cams. It should be noticed that the last image of this figure is an example of the constant-breadth cam obtained by CNC milling.

Different machining phases were required, such as: (1) Cutting off of the starting cylinder (aluminium block 6082, with a purity of 96%). (2) Face milling; contouring of the hub; contouring the cam profile; marking, drilling and keyway slotting. (3) Cutting off the contoured cam to define its thickness. (4) Face milling of the lower surface of the cam, leaving a total length of 17.40 mm. (5) Manual filing of the cam edges, removing any sharp edges. Table 3 shows the tools, fixture system and cutting conditions employed to obtain the metallic cams. The dimensions of the keyway and the length of the hub were modified to adapt them to the tools available in the workshop.

### 2.3. Measuring Process of the Cams

**Figure 13:**
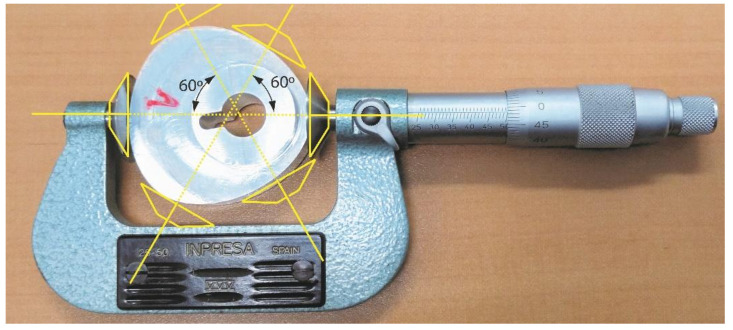
Dimensional measurement of the machined cams using a flange outside micrometre, and measuring directions.

The dimensional measurements of both the 3D-printed and the machined cams were carried out with a conventional disk flange outside micrometre, with a range of 25–50 mm and a precision of 0.01 mm. Figure 13 shows a measuring example for a metallic cam. Three measurements were carried out on each cam, in the three directions indicated with yellow dotted lines in the figure. It should be noticed that parallel micrometre discs (also indicated in yellow colour) are always tangent to the cam profile.

The relative error (*RE*) is calculated as follows:

(8) $$RE\;\left(\%\right)=[\frac{\mathrm{Measured}\;\mathrm{value}-\mathrm{Theoretical}\;\mathrm{value}}{\mathrm{Theoretical}\;\mathrm{value}}]\cdot 100$$

**Figure 14:**
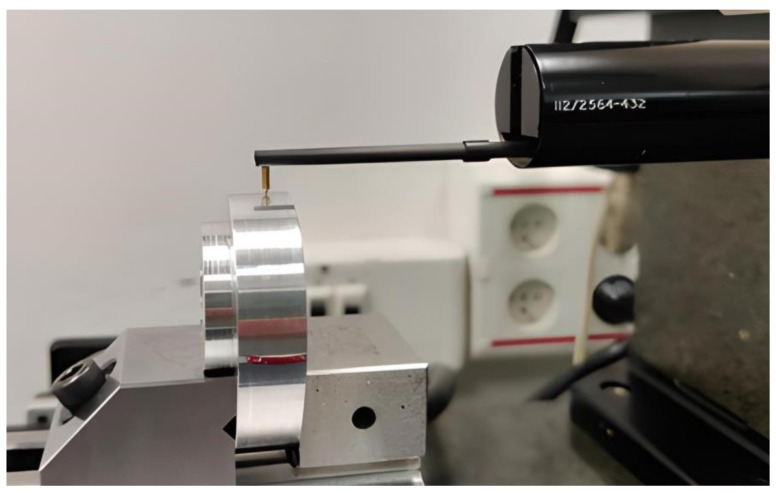
Roughness measurement of the working face of a machined cam.

The surface roughness of the working face of the cam that is in contact with the follower was measured with a Taylor Hobson Talysurf v2 contact roughness meter, using a Gaussian filter with a cut-off value of 0.8 mm. The 3D-printed and metallic cams were measured by means of the same process. Three measurements were taken on each cam—each one in a different area of the working face, in the longitudinal direction. Figure 14 depicts the case of a machined cam.

## 3. Results

In the following subsections, the results for the dimensions and roughness of the cams are presented.

### 3.1. Dimensions

The dimensions of both the 3D-printed and the machined cams are presented in Table 4. It should be noticed that the design parameter constant-breadth *d*_c_ of the cam is the principal dimension (Figure 8), which determines the proper assembly and correct operation of this type of mechanism.

Table 4 shows that a similar relative error is obtained in the 3D-printing process and the machining process when diametral dimensions, for example, *d*_c_ and *d*_ic_, are considered (which depends on x and y axes in the printing machine). However, when the z dimension is considered–for example, in this case the parameters *l* and *h*—a much higher error is obtained in the 3D-printing process than in the machining process. This can be attributed to the fact that, in the 3D-printed cams, the z dimension greatly depends on the conditions in which the subsequent layers are deposited. The layer height is one of the main printing parameters influencing surface roughness. In this case, the value of this parameter used for printing the cams was 0.15 mm (Table 2). 

### 3.2. Surface Roughness

**Figure 15:**
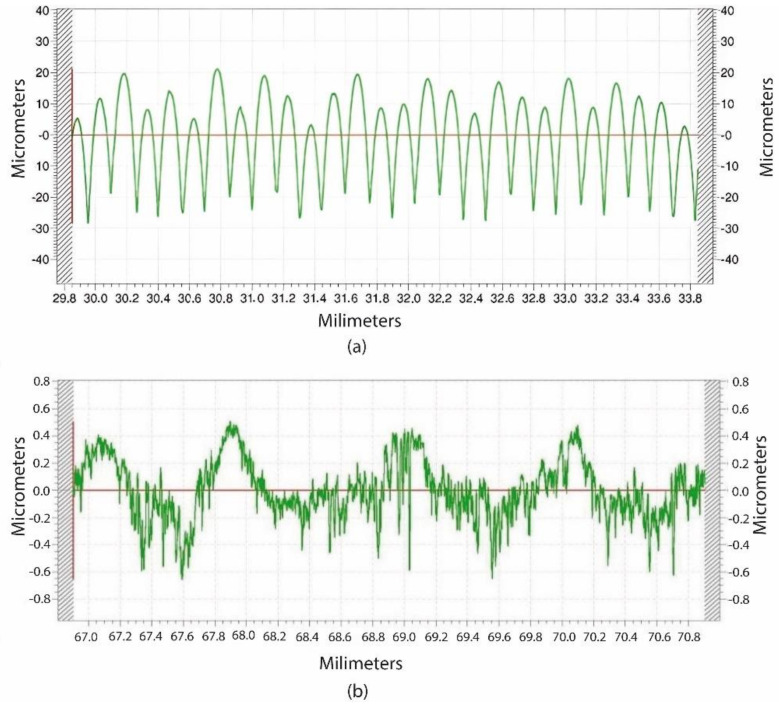
Roughness profile graphs of (**a**) a printed cam and (**b**) a milled cam.

Figure 15 shows two examples of roughness profile graphs and the corresponding values of the surface roughness measurements, for both the 3D-printed cam (Figure 15a) and the milled cam (Figure 15b), respectively. Table 5 contains the *R*_a_ values, as well as the standard deviation, for both the 3D-printed and the machined cams.

As mentioned in Section 2.3, roughness was measured along the longitudinal direction of the cam, which corresponds to a generatrix of the working face of the cam. From Figure 15a, it can be seen that the 3D-printed cam has a regular roughness profile, with rounded peaks and sharp valleys, in which each peak corresponds to a printed layer. On the contrary, from Figure 15b it is observed that, for the machined cam, a more irregular profile is obtained along the machined furrow. This is usual when a side milling operation is employed [24]. Only small peaks and valleys are detected, corresponding to slight machining marks.

By analysing the results shown in Table 5, the *R*_a_ values for the machined cams were 20 times lower than for the 3D-printed ones. It is well known that, in FFF processes, high surface roughness is obtained in lateral walls, which are perpendicular to the deposition plane of the layers [25]. Similar *R*_a_ values up to 0.41 µm were previously reported in machined flank cams [9].

## 4. Discussion

The relative error values achieved in this work for 3D-printed cams are similar to those obtained for 3D-printed gears in PLA material in the *xy* plane (below 0.8 %) [26]. These errors are of the same order of magnitude as the relative errors of the machined parts. However, higher relative errors of up to 1.64% were obtained for 3D-printed cams in the Z direction, which is perpendicular to the deposition plane of the parts. Beniak et al. [27] found that high layer height and high printing temperature led to lower dimensional accuracy in 3D-printed parts. Pennington et al. [28] observed that part size and printing temperature influenced dimensional accuracy in 3D-printed parts. It is not only the 3D printing parameters that influence dimensional accuracy, but also other parameters such as the material type, the material composition and the part geometry [29]. To reduce the dimensional error of PLA-printed parts containing cylindrical holes, Popescu et al. proposed using a high layer height of 0.32 mm, three shells and a high printing speed of 65 mm/s as the 3D printing parameters [30].

As for the requirements regarding dimensional accuracy in metallic cams, Rothbart [31] defined, in a milled and ground cam, an accuracy of ± 0.0254 mm for an approximate diameter of 300 mm in a cylindrical cam. In this work, a higher value of ± 0.06 mm is reported, considering only the milling operation. 

Although in this work the cams were manufactured by means of milling operations and no grinding was carried out, average roughness *R*_a_ was approximately 20 times lower for the machined parts than for the 3D printed ones. This highlights one of the main disadvantages of the FFF technology, due to the fact that the parts are manufactured layer-by-layer [27]. In FFF processes, roughness mainly depends on the selected layer height. Although this parameter could be reduced, it is limited and the quality of the parts. Specifically, the dimensional accuracy would worsen if a too low layer height value were selected [32]. In the case here presented, a value of 0.15 mm was used, providing a good quality of the 3D printed cams. Galantucci et al. [33] reported that roughness depends on nozzle diameter, raster width and layer height. Hartcher-O’Brien et al. [34] found that low layer height and low print speed favour low surface roughness.

Regarding metallic cams, the required surface finish is Ra < 0.4 µm [35]. This often implies a subsequent grinding operation [36]. In aluminium machined cams, Veera et al. [37] reported Ra values around 1 µm, which are higher than those obtained in this work. For structural steel flank cams, lower Ra values below 0.3 µm were reported [9].

According to the results in the present work, and considering the dimensional accuracy, it would be possible to replace metallic cams with plastic ones in mechanisms that have more of a kinematic type of operation than of high-power transmission. On the other hand, for that kind of mechanism, the surface finish is a less significant influential factor regarding the proper operation of the mechanism.

## 5. Conclusions

In the present work, constant-breadth cams were designed, manufactured and measured. In the design process, the QtCAM software was used to obtain and verify the cam profile, and the SolidWorks software was used to create the virtual model of the cam–follower mechanism and validate its correct operation. Regarding the design process, the main conclusion is:

- Two principal design desmodromic constraints must be considered in the design process of a constant-breadth cam that drives a double flat-translating follower. The first constraint is that the motion law *s*(θ) consists of two segments: the designed segment, which can only be designed in the interval from 0 to 180° of cam rotation), and the calculated segment, which is obtained using the expression 1. The second constraint is that the value of the constant-breadth cam *d*_c_ is equal to the diameter of the base circle plus the maximum displacement of the cam follower.

In the manufacture of the cams, two different processes were used: extrusion 3D printing utilizing the fused filament fabrication (FFF) technology and CNC machining. Both the dimensions and the surface finish of the cams were measured. The main conclusions are as follows:

- Similar dimensional relative errors were obtained for the 3D-printed cams and the machined parts when the *x*, *y* plane was considered (maximum relative error of 0.75%). However, when the *z* direction was considered, which is orthogonal to the depositing plane of the printed layers, higher dimensional relative errors were reported for the 3D-printed cams (up to 1.64%) than for the machined ones (up to 0.54 %);
- As for the surface finish on the lateral surface (working face) of the cams, the average roughness *R*_a_ was around 20 times higher for the 3D-printed cams (10.41 µm) than for the machined ones (0.50 µm).

According to the results obtained in this work, it would be possible to temporally replace metallic cams with plastic ones in mechanisms that have more of a kinematic type of operation than of high-power transmission. Little information is available about the dimensional accuracy and surface finish of 3D-printed desmodromic cams. Thus, this work will contribute to the study and analysis of this kind of mechanism.

## Figures and Tables

**Table 1:** Geometric parameters chosen for the design of the cam–follower mechanism.

Geometric Parameter [Units]	Numerical Value
Radius of the base circle of the cam $R_{b}$ [mm]	17.5
Breadth of the cam $d_{c}$ [mm]	50
Offset of the translating follower *ε* [mm]	0
Inclination angle of the translating follower *β* [°]	0

**Table 2:** Main 3D-printing parameters for the cams.

Parameter [Units]	Numerical Value
Nozzle diameter [mm]	0.4
Infill ratio [%]	18
Print speed [mm/s]	60
Infill pattern	Linear
Shell thickness [mm]	0.4
Layer height [mm]	0.15
Printing head temperature [°C]	200
Build plate temperature [°C]	60
Raster angle [°]	45
Number of wall lines	3

**Table 3:** Characteristics of the machining operations to obtain the metal cams.

Operation Number	Operation (Machine Tool)	Tool	Fixture System	Cutting Conditions
1	Cutting off of the starting cylinder (band saw machine)	Band saw	Bench vice	Cutting speed: 30 m/min
2	Face milling (CNC machine centre)	Face milling tool of diameter 40 mm and z = 8	Bench vice	Cutting speed: 150 m/min; feed speed: 955 mm/min; depth of cut: 2 mm
3	Rough contour milling of the cam hub (CNC machine centre)	Face milling tool of diameter 25 mm and z = 4	Bench vice	Cutting speed: 120 m/min; feed speed: 611 mm/min; depth of cut: 5.40 mm
4	Rough contour milling of the cam profile (CNC machine centre)	Face milling tool of diameter 25 mm and z = 4	Bench vice	Cutting speed: 120 m/min; feed speed: 611 mm/min; depth of cut: 20 mm
5	Marking of the cam hole (CNC machine centre)	Drilling tool of angle 90° and diameter 12 mm and z = 2	Bench vice	Cutting speed: 95 m/min; feed speed: 302 mm/min; depth of cut: 5 mm
6	Drilling of the cam hole (CNC machine centre)	Drilling tool of angle 120° and diameter 12 mm and z = 2	Bench vice	Cutting speed: 95 m/min; feed speed: 302 mm/min; cutting length: 22 mm
7	Contour milling of the keyway of 6 × 4.36 mm (CNC machine centre)	Face milling tool of diameter 4 mm and z = 2	Bench vice	Cutting speed: 120 m/min; feed speed: 1910 mm/min; depth of cut: 22 mm
8	Contour milling of the cam profile (CNC machine centre)	Face milling tool of diameter 10 mm and z = 4	Bench vice	Cutting speed: 120 m/min; feed speed: 1528 mm/min; depth of cut: 15 mm
9	Contour milling of the cam’s hub (CNC machine centre)	Face milling tool of diameter 10 mm and z = 4	Bench vice	Cutting speed: 120 m/min; feed speed: 1528 mm/min; depth of cut: 5.40 mm
10	Cutting off the machined cylinder (band saw machine)	Band saw	Bench vice	Cutting speed: 30 m/min
11	Face milling to final length of the cam of 17.40 mm, obtaining a width of the working face of 12 mm (CNC machine centre)	Face milling tool of diameter 40 mm and z = 8	Bench vice	Cutting speed: 150 m/min; feed speed: 955 mm/min; depth of cut: 2 mm

**Table 4:** Dimensions of the 3D-printed and machined cams.

Cam Manufacturing Process	Parameter	Theoretical Value (mm)	Mean Value (mm)	Standard Deviation (mm)	Relative Error (%)
3D-printed	*d*_c_	50	49.98	0.06	0.12
	*d*_ic_	12	11.91	0.05	0.75
	*l*	15	15.17	0.01	1.16
	*h*	12	12.20	0.01	1.64
Machined	*d*_c_	50	49.99	0.06	0.07
	*d*_ic_	12	11.97	0.01	0.30
	*l*	17.40	17.31	0.08	0.54
	*h*	12	11.99	0.09	0.08

**Table 5:** Average surface roughness *R*_a_ for the 3D-printed and machined cams.

Cam Manufacturing Process	Mean Value [µm]	Standard Deviation [µm]
3D-printed cams	10.41	0.12
Machined cams	0.50	0.03

## Data Availability

Data are available upon request.
